# Supplementary material for: Mdivi-1 Induced Mitochondrial Fusion as a Potential Mechanism to Enhance Stress Tolerance in Wheat
Source: Life (Basel). 2022 Sep 6;12(9):1386. doi: 10.3390/life12091386 (PMC9503966; doi:10.3390/life12091386)
Supplement: Supplementary file 1 [file life-12-01386-s001.zip › life-1906648-supplementary.pdf]

Table S1. Primers used for qPCR

Primer name*	Sequence 5'–3'	Product size, bp	Tm, °C
ARF	F: GCTCTCCAACAACATTGCCAAC<br>R: GCTTCTGCCTGTCACATACGC	165	55
RLI	F: CGATTCAGAGCAGCGTATTGTTGC<br>R: GCCTGTAGTTGGTCGGGTCTCTTC	242	60
ATG8 af	F: CGCATAAGGGAGAAGTACTCTGACA<br>R: CAAAGATGAAGATCGCCTTCTCA'	181	60
ATG8 g	F: GGCTGATAAGTCTGATGTCCCG<br>R: GAAGCAGTCGGTGGCAAGG	198	65
ATG8 h	F: GGTTCCGTGTGACATGCCA<br>R: GCCATCCTGCTTATCTTTGTACG	161	60
SNRK beta/gamma subunit	F: CCAATCATCTATTCATCGTCA<br>R: CGCAACATAGCCAATGGAT	209	60
SNRK beta 3 subunit	F: GGCAGGGATGACCATGAA<br>R: CGGGAAGCTCAGCAGTGT	156	60
SNRK beta 1/2 subunit 1L chromosome	F: CGGATGAATCCACCAACAAT<br>R: GACCTCTCTAACACCTTCCTTGA	133	60
SNRK beta 1/2 subunit 4L chromosome	F: GTTCCTGAAAGTGTTGAAAGTGT<br>R: GGTGGTTGAGGACGATGTG	199	60
SNRK alpha subunit 1L chromosome	F: CAGTATGGTTCTCCAGGGTTT<br>R: GCCATCAGCTTCAATTATAGCA	255	60
SNRK alpha subunit 3L chromosome	F: GGAAATCGGCAACAACCATA<br>R: CCAGGTCGTCGGTTTCAATA	277	60

* For abbreviations of genes see Materials and Methods.
